# Occurrence and types of medication error and its associated factors in a reference teaching hospital in northeastern Iran: a retrospective study of medical records

**DOI:** 10.1186/s12913-022-08864-9

**Published:** 2022-11-28

**Authors:** Seyed Saeed Tabatabaee, Vahid Ghavami, Javad Javan-Noughabi, Edris Kakemam

**Affiliations:** 1 Social Determinants of Health Research Center, Mashhad University of Medical Sciences, Mashhad, Iran; 2 Department of Health Economics and Management Sciences, School of Health, Mashhad University of Medical Sciences, Mashhad, Iran; 3 Department of Biostatistics, School of Health, Mashhad University of Medical Sciences, Mashhad, Iran; 4 Clinical Research Development Unit of Tabriz Valiasr Hospital, Tabriz University of Medical Sciences, Tabriz, Iran

**Keywords:** Nurses, Medication errors, Hospitals, Teaching

## Abstract

**Background:**

Medication errors are categorized among the most common medical errors that may lead to irreparable damages to patients and impose huge costs on the health system. A correct understanding of the prevalence of medication errors and the factors affecting their occurrence is indispensable to prevent such errors. The purpose of this study was to investigate the prevalence and types of medication errors among nurses in a hospital in northeastern Iran.

**Methods:**

The present descriptive-analytical research was conducted on 147 medical records of patients admitted to the Department of Internal Medicine at a hospital in northeastern Iran in 2019, selected by systematic sampling. The data were collected through a researcher-made checklist containing the demographic profiles of the nurses, the number of doctor's orders, the number of medication errors and the type of medication error, and were finally analyzed using STATA version 11 software at a significance level of 0.05.

**Results:**

Based on the findings of this study, the mean prevalence of medication error per each medical case was 2.42. Giving non-prescription medicine (47.8%) was the highest and using the wrong form of the drug (3.9%) was the lowest medication error. In addition, there was no statistically significant relationship between medication error and the age, gender and marital status of nurses (p > 0.05), while the prevalence of medication error in corporate nurses was 1.76 times higher than that of nurses with permanent employment status (IRR = 1.76, p = 0.009). The prevalence of medication error in the morning shift (IRR = 0.65, p = 0.001) and evening shift (IRR = 0.69, p = 0.011) was significantly lower than that in the night shift.

**Conclusion:**

Estimating the prevalence and types of medication errors and identified risk factors allows for more targeted interventions. According to the findings of the study, training nurses, adopting an evidence-based care approach and creating interaction and coordination between nurses and pharmacists in the hospital can play an effective role in reducing the medication error of nurses. However, further research is needed to evaluate the effectiveness of interventions to reduce the prevalence of medication errors.

## Introduction

Medication error is one of the basic problems of health systems all over the world, which can be a serious threat to the safety of patients [1, 2]. Medication errors can lead to unpleasant consequences such as prolonged hospitalization, increased treatment costs and even death [3]. The results of a survey conducted in 2018 in the UK found that more than 2 million people are affected by the complications of medication error every year, and give rise to death almost 100 thousand people [4]. Medication error is the third leading cause of death in America [5]. In addition to weakening patients' trust in medical services and the health care system, medication errors can impose huge costs on the health sector [6]. The findings of a previous study reported that the cost imposed per medication error ranges from €2.58 to €111 727.08 [7].

Medication error refers to any malpractice in the medication process (prescribing, preparing and giving medication to the patient), regardless of whether it has side effects for the patient or not, which can occur at any stage of the drug therapy cycle from prescription, transcription, distribution to drug administration [7]. However, previous studies have shown that most errors occur when the medication is delivered to the patient [8]. Doctors' prescriptions, nurses' implementation of drug orders, pharmacists' reading of prescriptions in pharmacies, and sometimes patients themselves and their families play a role in medication errors [9]. In addition, medication errors are more common in hospitals when implementing medication orders [10]. Such evidence suggests that all health care professionals, including physicians, nurses, and pharmacists, are involved in such errors. However, a review study in Iran documented that the highest prevalence of medication errors was among nurses and nursing students [11]. This is because nurses mostly implement drug orders and drug therapy is the most common treatment and care intervention performed by nurses [12]. Many factors are implicated in such events, including high workload and overtime [13]. Nurses themselves believe that the main causes of medication error can be the use of abbreviations instead of the full names of drugs, similarity in the names of drugs, carelessness of nurses, high work pressure and workload especially in emergency situations, low drug information and weakness in continuous education [3].

Some studies have examined the medication errors in Iran. For instance, a recent systematic and meta-analysis study on 8 studies reported that the overall medication error’s prevalence among nursing students was 39.68% and the prevalence of lack of reporting medication errors was 48.60% [14]. Likewise, another systematic review on 40 eligible articles about medication errors in Iran showed that the prevalence of the medication error ranged from 10 to 80% [11]. Karami Matin et al. in a systematic review and meta-analysis on the 22 studies with 3556 samples showed that the prevalence of medication errors among nurses in hospitals in Iran were 53% with a confidence interval of 95% between 41%-60% [15]. Although accurate statistics of the consequences of medication errors were not found in Iran, annual costs for prolonged hospitalization and extra care due to medication errors exceeded billions of Tomans (Iran’s currency) [16–18].

Although medication error is a critical clinical problem that seriously threatens the safety of patients, studies have shown that almost half of these cases can be prevented by implementing simple standards [18]. To achieve this goal, the third generation of the National Accreditation Program has paid special attention to patient safety, and one of the eight axes of this program is dedicated to the management of drugs and equipment. Thus, the drug management consists of nine standards, including ensuring the hospital's access to drugs, safe storage of drugs, prescription drugs, supply and distribution of drugs, management of drug consumption, continuous evaluation of the process of prescription and drug consumption, monitoring of expired drugs, review of drugs prescribed and medication error control [19, 20].

Although positive measures have been taken to prevent and control medication error, it is still a big challenge in Iran. A systematic review study documented that the prevalence of medication error in the countries of the Middle East region, including Iran, varies between 7 and 90% [21]. Therefore, correct identification of type of medication errors is an important step in preventing their recurrence. According to the review of the literature, most of the studies conducted in the field of prevalence and types of medication errors in Iran have only examined the opinions and experiences of nurses [22, 23]. Therefore, the current study was conducted to investigate the prevalence and type of medication errors by nurses on the medical records of patients admitted to the Department of Internal Medicine at a hospital in northeastern Iran.

## Methods

### Study design and setting

The present cross-sectional and descriptive-analytical study was conducted in 2019 in a referral teaching hospital in the northeast of the country. The hospital operates in various fields of treatment, education and research, with 776 active beds and 46 wards. Currently, 2950 medical personnel including nurses, doctors, paraclinical, support and administrative personnel are working in this hospital. Among the inpatient departments, the Department of Internal Medicine with 61 active beds with an occupancy rate of 83% and due to the large volume of medication orders compared to other wards was selected as the study setting.

### Study population and sample

The research population included all the medical records of patients admitted to the Department of Internal Medicine at the mentioned hospital (n = 6995), among which 147 cases were selected by systematic sampling method and based on the following equation, taking into account the error level (α) of 0.05, accuracy (d) of 0.08 and limited population size (N).

### Data collection tool

The data were collected through a researcher-made checklist containing the demographic profiles of the nurses (age, gender, marital status, type of employment, shift work and work experience), the number of doctor's orders, the number of medication errors and the type of medication error (giving the wrong medicine, wrong medicine dose, giving medicine at the wrong time, not giving medicine, drug interaction, etc.). Employment type is included permanent, contract, corporative, and bonded. Corporate forces are forces that are hired by intermediary companies (human resources supply companies) for government organizations. The intermediary company receives the salaries and benefits of the individuals from the relevant main organization and pays to its corporate forces. Therefore, corporate forces do not have a direct financial relationship with government institutions.

A number of nursing experts and quality improvement officers confirmed validity of the checklist. The reliability of the checklist was supported by the findings of an internal consistency reliability. The Cronbach's alpha coefficient for all items was greater than 0.7.

### Data collection

To do this, first, the necessary permits were obtained from the deputy of research of the university and the hospital directorate in cooperation with the nursing management of the hospital. Then, two trained nurses went to the medical records unit, studied the nursing reports and matched the report with the doctor's order report. Medication error was identified and recorded through non-compliance of nursing report and doctor's orders. In order to increase the accuracy in identifying medication errors, each medical file was reviewed by two nurses separately and any contradictions in the collected information were resolved on the spot and by mutual consultation and third person judgment. The hospital matron and two nurse managers with a history of membership in the medication errors committee were selected to collect data. Despite the familiarity of the selected nurses with medication errors, three two-hour sessions were held for the training of nurses about types of medication errors. The inclusion criteria were the existence of a file in the sector of medical records and also the sector of hospitalization process at the time of discharge from the Department of Internal Medicine. The exclusion criteria included lack of access to files. It should be noted that the patient file information was kept confidential.

### Data analysis

After completing the checklist, the demographic characteristics of the nurses noted in the checklist were extracted through the personnel system. Descriptive statistics were used to analyze the data, the mean and standard deviation for quantitative variables as well as frequency and percentage for qualitative variables. Since the nurses' performance was error-free in many cases, Poisson and classical negative binomial regression models were not suitable, and so Zero Inflated Poisson (ZIP) and Zero Inflated Negative Binomial (ZINB) regression models were fitted to these zero inflated data [24, 25]. On the other hand, if there is over-dispersion in the data, the ZINB model will be preferred to the ZIP model, therefore, Likelihood-Ratio Test (LRT) was used to check over-dispersion in the data [26]. Because there was more than one data record for each nurse, robust standard errors were estimated for the regression coefficients. All data were finally analyzed using STATA version 11 software at a significance level of 0.05.

## Results

**Fig. 1:**
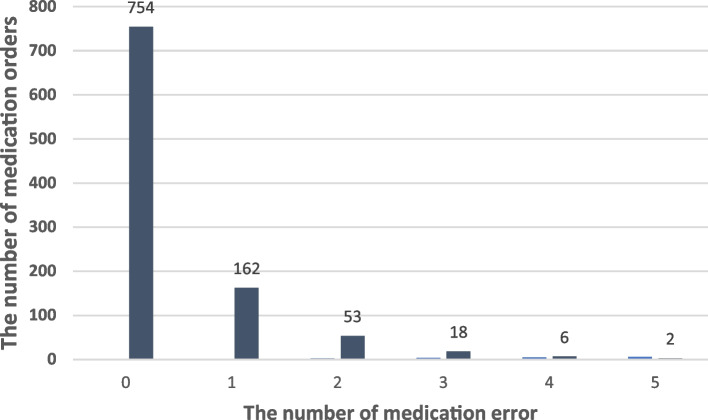
Distribution of nurses' medication errors

This study investigated the performance of 57 nurses who had implemented 955 doctors' orders. The distribution of the number of medication errors is shown in Fig. 1. One of the important features of the number of errors in this study was the zero-medication error in 754 doctor's orders (76%).

Regarding the type of errors, giving non-prescription medicine (47.8%) was the highest and using the wrong form of the drug (3.9%) was the lowest medication error (Table 1).

**Table 1:** Type of medication errors

Type of medication error	Frequency	Percentage
giving medicine at the wrong time	20	5.6
giving the wrong drug to the patient	33	9.3
using the wrong form of the drug	14	3.9
failure to consider drug interactions	61	17.1
wrong dose	25	7.0
giving non-prescription medicine	170	47.8
not giving the prescribed drug (omission)	33	9.3
Total	356	100

The average age of the nurses was 34 ± 7 years, the average work experience was 7 ± 2.5 years, and the majority of nurses were female (70%). The majority of nurses (66%) were married. Other demographic information of nurses is shown in Table 2.

**Table 2:** Demographic characteristics of nurses

Variable	Frequency	Percentage
**Gender**		
* Female*	40	70
* Male*	17	30
**Marital status**		
* Single*	19	33.33
* Married*	38	66.67
**Employment type**		
* Contract*	4	7.02
* Corporative*	14	24.57
* Bonded*	20	35.08
* Permanent*	19	33.33
	**Mean ± Standard deviation**	
**Age**	34 ± 7	
**Work experience**	7 ± 2.5	

Due to the high number of cases without errors in medication orders (Fig. 1), ZIP and ZINB regression models were fitted to the data. LRT results showed over-dispersion in the data (χ^2^ = 6.2 and p = 0.01), so ZINB was determined as the final model. The fitting results of the ZINB model are reported in Table 3.

**Table 3:** The results of fitting the Zero Inflated Negative Binomial (ZINB) regression model on the number of medication errors of nurses

Variable	IRR^*^(%95 CI)	P-value
**Age**	0.98 (0.1–96.01)	0.250
**Gender** (reference: *Male*)	0.99 (0.1–7.39)	0.951
**Marital status** (reference: *Single*)	1.21 (0.1–85.71)	0.294
**Employment type (**reference**:** *Permanent***)**		
*Contract*	1.08 (0.1–60.96)	0.789
*Corporative*	1.76 (1.2–15.69)	*0.009*
*Bonded*	0.73 (0.1–45.19)	0.210
**Shift work** (reference: *Night*)		
*Morning*	0.65 (0.0–5.85)	*0.001*
*Evening*	0.69 (0.0–52.92)	*0.011*

*Incidence Rate Ratio

Based on the results of ZNIB model, the prevalence of medication error in corporate nurses was 1.76 times higher than that of nurses with permanent employment status (IRR = 1.76, p = 0.009). Moreover, the prevalence of medication error in the morning shift (IRR = 0.65, p = 0.001) and evening shift (IRR = 0.69, p = 0.011) was significantly lower than that in the night shift.

## Discussion

The aim of the present study was to estimate the prevalence and types of medication error in a referral teaching hospital in the northeast of Iran. The findings of the present study indicated that a large part of all medication orders (76%) had no medication errors, and among the medication orders with errors, 67% had only one error. The average prevalence of medication error per nurse in this study was estimated to be 6.1, which is lower than the average of other numerical studies. In a study that examined medication error in the emergency and pediatric departments of 24 teaching hospitals in the capital of Iran, Tehran, the average total medication error per nurse was estimated to be 41.9 [27]. This big difference can be attributed to the selection of emergency and pediatric departments as the research community and therefore the high workload of these two wards. In a similar study in Jordan, the average medication error per nurse was reported to be 2.2 [28]. In the current study, the average medication error per case/patient was 2.42, indicating that on average 2–3 medication errors occurred for each patient during the study period. In a similar study in the south of Iran, the prevalence of medication error was assessed through a hidden direct observation method by a trained observer. The results of that study showed that 96.5% of patients experienced at least one medication error and 3.5 medication errors were reported per patient [29], which is similar to the statistics obtained from the present study.

However, in other studies in Iran and India, the above index was obtained as 1.3 and 0.15, respectively, which is lower than the present study [30, 31]. One of the reasons for the difference in findings can be the lack of a clear definition for medication error, as well as the variety of medication error identification and measurement methods used in different studies.

Analyzing the type of medication error in the current study showed that almost half of the medication error (47.8%) occurred during the study period was the type of giving medicine (by the nurse) to the patient without a doctor's prescription, which can also be referred to as the "wrong drug" error. After that, failure to consider drug interactions (17.1%), not giving the prescribed drug (omission) and giving the wrong drug to the patient (9.3%) are common forms of medication error. It seems that training nurses, adopting an evidence-based care approach and creating interaction and coordination among physicians and nurses and exchanging information about new drugs, making available pharmacology books in clinical departments, holding pharmacology conferences and attending full time pharmacists and pharmacologists in the hospital can play an effective role in reducing such errors.

Findings of Mulac et al. (2021) in Norway in a study using a modified version of the WHO Conceptual Framework for the International Classification for Patient Safety showed that the main types of medication errors in the studied hospitals were dosing errors (38%), omissions (23%) and wrong drug (15%) [32]. In another study, the most common type of medication error has been identified as the wrong dose and wrong administration [33]. In a study by Gebre et al. in Ethiopia, the main types of medication error were reported in the wrong drug, wrong dose and monitoring error [34]. The results of a study in Iran that examined medication error from the nurses' point of view showed that giving medicine at the wrong time, giving several oral medicines at the same time and giving painkillers after surgery without a doctor's prescription were the three most frequent types of medication error [22]. The findings of the present study are consistent with the mentioned results.

Examining the association between some factors and medication error in the present study showed no statistically significant relationship between medication error and the age, gender and marital status of nurses. This finding is consistent with the similar study, in which no significant relationship was observed between medication error and the two parameters of age and gender [33]. The results of some studies confirm the relationship between gender and medication error, for example, one study reported a higher prevalence of medication error in male nurses [27]. In this study no statistically significant relationship was found between the number of medication errors and the parameters of age and marital status [27], which is consistent with the results of the current study.

According to the research findings, the employment status of nurses is an important factor in the occurrence of medication error, so that the probability of medication error in corporate nurses was calculated to be 1.76 times more than that of permanent nurses, which could be due to the less work experience of corporate nurses and lack of job stability. Different studies have also reported a significant relationship between low work experience and medication error; for example, one study reported the highest medication error in nurses with less than 5 years of work experience [33]. Another study found no significant relationship between the employment status and the medication error [27].

In the present study, the prevalence rate of medication error in the morning and evening shifts was significantly lower than that in the night shift, which can be attributed to the sleepiness of the personnel and the subsequent decrease in their productivity. Previous studies also showed that the number of medication errors of nurses in night shifts was higher than in morning shifts [27], which is consistent with the results of the current study. Other studies also concluded that the probability of medication error occurring in night shifts is higher due to sleepiness of employees or shortage of personnel [29, 35, 36].

## Study limitations

One of the limitations of the present study was the inaccessibility to the functional characteristics of nurses such as education, experience, as well as job components such as work pressure and working hours. Another limitation of the present study was the use of patients' medical records and conducting the study only in one hospital ward, which limits the generalizability of the findings.

## Conclusions

The present study showed that giving medicine to the patient without a doctor's prescription and ignoring drug interactions had the highest prevalence of medication error. In addition, corporate employment status and night shift were the most important factors affecting on medication error. Therefore, preparing guidelines and training nurses about paying attention to doctor's orders and drug interactions can play an effective role in reducing such errors. Also, the use of experienced nurses alongside corporate nurses with less work experience and further supervision of nurses in the night shift can be effective in preventing medication errors.

## Data Availability

The datasets generated and/or analysed during the current study are not publicly available confidentiality of information but are available from the corresponding author on reasonable request.

## Abbreviations

ZIP: Zero Inflated Poisson
ZINB: Zero Inflated Negative Binomial
LRT: Likelihood-Ratio Test

## References

[1] Alqenae FA, Steinke D, Keers RN (2020). Prevalence and nature of medication errors and medication-related harm following discharge from hospital to community settings: a systematic review. Drug Saf.

[2] Kakemam E, Albelbeisi AH, Davoodabadi S, Azarmi M, Zolghadr F, Mamene M (2022). The impact of nurses' perceptions of systems thinking on occurrence and reporting of adverse events: A cross-sectional study. J Nurs Manag.

[3] Ghasemi F, Babamiri M, Pashootan Z (2022). A comprehensive method for the quantification of medication error probability based on fuzzy SLIM. PLoS ONE.

[4] Parekh N, Ali K, Stevenson JM, Davies JG, Schiff R, Van der Cammen T (2018). Incidence and cost of medication harm in older adults following hospital discharge: a multicentre prospective study in the UK. Br J Clin Pharmacol.

[5] Ahmad FB, Anderson RN. The leading causes of death in the US for 2020. JAMA. 2021;325(18):1829-30. 10.1001/jama.2021.5469 PMC8145781 33787821

[6] Elliott R, Camacho E, Campbell F, Jankovic D, St James MM, Kaltenthaler E et al. Prevalence and economic burden of medication errors in the NHS in England. Rapid evidence synthesis and economic analysis of the prevalence and burden of medication error in the UK. 2018

[7] Walsh EK, Hansen CR, Sahm LJ, Kearney PM, Doherty E, Bradley CP (2017). Economic impact of medication error: a systematic review. Pharmacoepidemiol Drug Saf.

[8] Kim KS, Kwon SH, Kim JA, Cho S (2011). Nurses’ perceptions of medication errors and their contributing factors in South Korea. J Nurs Manag.

[9] Warholak TL, Queiruga C, Roush R, Phan H. Medication error identification rates by pharmacy, medical, and nursing students. Am J Pharm Educ 2011;75(2):24. 10.5688/ajpe75224 PMC3073098 21519414

[10] Hanskamp-Sebregts M, Zegers M, Boeijen W, Westert GP, van Gurp PJ, Wollersheim H (2013). Effects of auditing patient safety in hospital care: design of a mixed-method evaluation. BMC Health Serv Res.

[11] Vaziri S, Fakouri F, Mirzaei M, Afsharian M, Azizi M, Arab-Zozani M (2019). Prevalence of medical errors in Iran: a systematic review and meta-analysis. BMC Health Serv Res.

[12] Rohde E, Domm E (2018). Nurses’ clinical reasoning practices that support safe medication administration: An integrative review of the literature. J Clin Nurs.

[13] Ofosu R, Jarrett P (2015). Reducing nurse medicine administration errors. Nurs Times.

[14] Dehvan F, Dehkordi AH, Gheshlagh RG, Kurdi A. The prevalence of medication errors among nursing students: A systematic and meta-analysis study. Int J Prev Med. 2021;12:21. 10.4103/ijpvm.IJPVM_418_19 PMC8106284 34084318

[15] Matin BK, Hajizadeh M, Nouri B, Rezaeian S, Mohammadi M, Rezaei S (2018). Period prevalence and reporting rate of medication errors among nurses in Iran: A systematic review and meta-analysis. J Nurs Manag.

[16] Karimian Z, Kheirandish M, Javidnikou N, Asghari G, Ahmadizar F, Dinarvand R (2018). Medication errors associated with adverse drug reactions in Iran (2015–2017): A P-method approach. Int J Health Policy Manag.

[17] Rezapour A, Javan-Noughabi J, Salehiniya H, Kassani A, Sadeghi A (2019). Albumin usage in Iran. Arch Iran Med.

[18] Javan-Noughabi J, Parnian E, Hajiesmaeili M, Salehiniya H, Setoodehzadeh F (2020). The impact of a guideline to prevent inappropriate albumin administration in a hospital in Iran. Br J Healthc Manag.

[19] Mosadeghrad AM, Ghazanfari F (2021). Developing a hospital accreditation model: a Delphi study. BMC Health Serv Res.

[20] Ebrahimipour H, Hooshmand E, Varmaghani M, Javan-Noughabi J, Mojtabaeian SM (2021). The challenges of physicians’ participation in hospital accreditation programs: a qualitative study in Iran. BMC Health Serv Res.

[21] Alsulami Z, Conroy S, Choonara I (2013). Medication errors in the Middle East countries: a systematic review of the literature. Eur J Clin Pharmacol.

[22] Piroozi B, Mohamadi-Bolbanabad A, Safari H, Amerzadeh M, Moradi G, Usefi D et al. Frequency and potential causes of medication errors from nurses’ viewpoint in hospitals affiliated to a medical sciences University in Iran. Int J Hum Rights Healthcare. 2019;12(4):267-75.

[23] Cheragi MA, Manoocheri H, Mohammadnejad E, Ehsani SR (2013). Types and causes of medication errors from nurse's viewpoint. Iran J Nurs Midwifery Res.

[24] Lambert D (1992). Zero-inflated Poisson regression, with an application to defects in manufacturing. Technometrics.

[25] Minami M, Lennert-Cody CE, Gao W, Román-Verdesoto M (2007). Modeling shark bycatch: the zero-inflated negative binomial regression model with smoothing. Fish Res.

[26] Agresti A. Categorical data analysis: Wiley. 2003

[27] Izadpanah F, Nikfar S, Imcheh FB, Amini M, Zargaran M (2018). Assessment of frequency and causes of medication errors in pediatrics and emergency wards of teaching hospitals affiliated to Tehran University of Medical Sciences (24 hospitals). J Med Life.

[28] Mrayyan MT, Shishani K, AL-Faouri I (2007). Rate causes and reporting of medication errors in Jordan: nurses’ perspectives. J Nurs Manag.

[29] Vazin A, Zamani Z, Hatam N (2014). Frequency of medication errors in an emergency department of a large teaching hospital in southern Iran. Drug Healthcare Patient Saf.

[30] Ahangar N, ALa S (2019). Evaluation of medication errors in internal wards of Imam Sajjad Ramsar Hospital In 2017 Spring and Summer. Med J Mashhad Univ Med Sci.

[31] Parthasarathi A, Puvvada R, Patel H, Bhandari P, Nagpal S. Evaluation of medication errors in a tertiary care hospital of a low-to middle-income country. Cureus. 2021;13(7):e16769. 10.7759/cureus.16769 PMC8328840 34354894

[32] Mulac A, Taxis K, Hagesaether E, Granas AG (2021). Severe and fatal medication errors in hospitals: findings from the Norwegian Incident Reporting System. Eur J Hosp Pharm.

[33] Zaree TY, Nazari J, Jafarabadi MA, Alinia T (2018). Impact of psychosocial factors on occurrence of medication errors among Tehran public hospitals nurses by evaluating the balance between effort and reward. Saf Health Work.

[34] Gebre M, Addisu N, Getahun A, Workye J, Gamachu B, Fekadu G (2021). Medication Errors Among Hospitalized Adults in Medical Wards of Nekemte Specialized Hospital, West Ethiopia: A Prospective Observational Study. Drug Healthcare Patient Saf.

[35] Shahrokhi A, Ebrahimpour F, Ghodousi A (2013). Factors effective on medication errors: a nursing view. Journal of research in pharmacy practice.

[36] Cloete L. Reducing medication errors in nursing practice. Cancer Nurs Pract. 2015;14(1):50-59. 10.7748/ns.29.20.50.e9507 25585768

